# Effects of Propranolol on Bone, White Adipose Tissue, and Bone Marrow Adipose Tissue in Mice Housed at Room Temperature or Thermoneutral Temperature

**DOI:** 10.3389/fendo.2020.00117

**Published:** 2020-03-17

**Authors:** Russell T. Turner, Kenneth A. Philbrick, Carmen P. Wong, Amanda R. Gamboa, Adam J. Branscum, Urszula T. Iwaniec

**Affiliations:** ^1^Skeletal Biology Laboratory, School of Biological and Population Health Sciences, Oregon State University, Corvallis, OR, United States; ^2^Center for Healthy Aging Research, Oregon State University, Corvallis, OR, United States; ^3^Biostatistics Program, School of Biological and Population Health Sciences, Oregon State University, Corvallis, OR, United States

**Keywords:** β-adrenergic, non-shivering thermogenesis, thermoneutral, cancellous bone, premature bone loss

## Abstract

Growing female mice housed at room temperature (22°C) weigh the same but differ in body composition compared to mice housed at thermoneutrality (32°C). Specifically, mice housed at room temperature have lower levels of white adipose tissue (WAT). Additionally, bone marrow adipose tissue (bMAT) and cancellous bone volume fraction in distal femur metaphysis are lower in room temperature-housed mice. The metabolic changes induced by sub-thermoneutral housing are associated with lower leptin levels in serum and higher levels of *Ucp1* gene expression in brown adipose tissue. Although the precise mechanisms mediating adaptation to sub-thermoneutral temperature stress remain to be elucidated, there is evidence that increased sympathetic nervous system activity acting via β-adrenergic receptors plays an important role. We therefore evaluated the effect of the non-specific β-blocker propranolol (primarily β_1_ and β_2_ antagonist) on body composition, femur microarchitecture, and bMAT in growing female C57BL/6 mice housed at either room temperature or thermoneutral temperature. As anticipated, cancellous bone volume fraction, WAT and bMAT were lower in mice housed at room temperature. Propranolol had small but significant effects on bone microarchitecture (increased trabecular number and decreased trabecular spacing), but did not attenuate premature bone loss induced by room temperature housing. In contrast, propranolol treatment prevented housing temperature-associated differences in WAT and bMAT. To gain additional insight, we evaluated a panel of genes in tibia, using an adipogenesis PCR array. Housing temperature and treatment with propranolol had exclusive as well as shared effects on gene expression. Of particular interest was the finding that room temperature housing reduced, whereas propranolol increased, expression of the gene for acetyl-CoA carboxylase (*Acacb*), the rate-limiting step for fatty acid synthesis and a key regulator of β-oxidation. Taken together, these findings provide evidence that increased activation of β_1_ and/or β_2_ receptors contributes to reduced bMAT by regulating adipocyte metabolism, but that this pathway is unlikely to be responsible for premature cancellous bone loss in room temperature-housed mice.

## Introduction

The thermoneutral zone, also called the zone of thermal comfort, is the temperature range where the resting rate of heat production is in equilibrium with the rate of heat loss to the environment. The thermoneutral range varies among species and is ~10°C higher for mice than for humans (1). Environmental temperatures outside the thermoneutral zone induce adaptive responses in homeothermic animals to preserve core body temperature (e.g., shivering to increase body temperature and sweating/panting to decrease body temperature). In contrast to humans and larger rodents such as rats, mice are not strict homeotherms, but instead function as daily facultative heterotherms. This fundamental difference in energy homeostasis contributes to important differences in physiology between mice and humans. Daily torpor, where decreases in body temperature result in energy savings, is a common strategy utilized by mice and other small mammals to cope with low environmental temperatures and fluctuations in food availability (2). Cold stress induced by housing mice at standard room temperature results in physiological changes that can influence experimental outcomes. Tumor growth, for example, is significantly greater in mice housed at room temperature than in mice housed at thermoneutrality, and anti-tumor immune response is suppressed at room temperature (3). Furthermore, chronic stress induced by housing mice at room temperature increases the resistance of hematopoietic stem and progenitor cells to total body radiation-induced apoptosis (4). The impact of cold stress on response to tumor growth and radiation in the aforementioned studies depends on β-adrenergic signaling and is antagonized by treatment with propranolol, a non-specific antagonist of β-adrenergic receptors (3, 4).

When housed at room temperature, C57BL/6 mice of both sexes exhibit rapid loss of cancellous bone prior to cessation of linear bone growth (5–7). Importantly, housing mice at thermoneutrality prevents the premature bone loss (6, 7). The lower cancellous bone volume fraction in distal femur of mice housed at room temperature compared to mice housed at thermoneutrality is due, in part, to decreased bone formation; osteoblast-lined bone perimeter, bone formation rate measured using fluorochrome labels, mRNA levels for bone matrix proteins, and serum osteocalcin are lower in mice housed at room temperature. In contrast, osteoclast-lined bone perimeter is higher, suggesting locally increased bone resorption (7). Bone formation and cancellous bone volume fraction are often inversely associated with bone marrow adiposity (8–15). However, an inverse relationship was not observed with housing temperature; female mice housed at room temperature had lower bone formation as well as lower bMAT compared to mice housed at thermoneutrality, whereas male mice had lower bone formation but did not exhibit differences in bMAT (6, 7).

Bone is innervated and bone growth and turnover balance is regulated, in part, via sensory and sympathetic signaling (16). β-adrenergic receptors are located in skeletal tissue (17) and treatment with propranolol is reported to influence bone mass in some animal models (17–21) and increase fat accrual in humans (22, 23). Thermogenesis induced by cold stress is mediated, at least in part, by increased sympathetic outflow from the hypothalamus to peripheral tissues, including brown adipose tissue (BAT) (24). The purpose of this study was to assess the role of β-adrenergic signaling (by blocking β-adrenergic receptors with propranolol) in mediating the differential effects of housing temperature on body composition, bone, and bMAT levels in female mice.

## Materials and Methods

### Experimental Design

The Institutional Animal Care and Use Committee approved the experimental protocol used in this study and animals were maintained in accordance with the NIH Guide for the Care and the Use of Laboratory Animals. A total of 40, 4-week-old, female C57BL/6 (B6) mice were obtained from Jackson Laboratory (Bar Harbor, ME, USA) and housed individually in a room on a 12 h light:12 h dark cycle. Food (Teklad 8604, Harlen Laboratories, Indianapolis, IN) and water were provided *ad libitum* to all animals. Body weight and food consumption were measured weekly for the 14-week duration of study.

Mice were randomized by weight into one of four groups, 22°C ± propranolol or 32°C ± propranolol (*n* = 10/group), and maintained at their respective temperatures and treatments until 18 weeks of age. Propranolol (Sigma, St. Louis) was administered in drinking water (0.5 g/l, pH 3.0) using aluminum foil-covered drinking tubes. Control mice received acidified water (vehicle). Water was changed twice/week. Water consumption was calculated as ml/d and the dose rate of propranolol calculated as mg/g/d. This method of delivery and dose of propranolol was chosen because it has been shown to be effective in blocking β_1_ and β_2_ but not β_3_ adrenergic receptors (25–27).

Mice were anesthetized with 2–3% isoflurane delivered in oxygen and body composition determined immediately prior to sacrifice. The mice were bled by cardiac puncture. Serum was collected and stored at −80°C for measurement of leptin and global markers of bone turnover. Abdominal white adipose tissue (WAT) and uteri were excised and weighed. Femora were removed, fixed overnight in 10% formalin, and stored in 70% ethanol for microcomputed tomography (μCT) and histomorphometric analyses. Tibiae and brown adipose tissue (BAT) were removed, frozen in liquid nitrogen, and stored at −80°C for mRNA analysis.

### Serum Chemistry

Serum leptin was measured using Mouse Leptin Quantikine ELISA Kit (R&D Systems, Minneapolis, MN), serum osteocalcin was measured using Mouse Gla-Osteocalcin High Sensitive EIA Kit (Clontech, Mountain View, CA), and serum CTX-1 was measured using Mouse CTX-1 ELISA kit (Life Sciences Advanced Technologies, Petersburg, FL) according to the respective manufacturer's protocol.

### Dual Energy X-Ray Absorptiometry

Percent body fat was determined using dual energy x-ray absorptiometry (DXA) (Piximus, Lunar Corp., Madison, WI, USA).

### Microcomputed Tomography

Bone volume and architecture were assessed using μCT. We scanned right femora in 70% ethanol using a Scanco μCT40 scanner (Scanco Medical AG, Basserdorf, Switzerland) at a voxel size of 12 μm on a side (55 kVp x-ray voltage, 145 μA intensity, and 200 ms integration time). We set filtering parameters sigma and support to 0.8 and 1, respectively. Bone segmentation was conducted at a threshold of 245 (scale, 0–1,000) determined empirically. Total femur mineralized tissue volume (cancellous + cortical bone) was evaluated first. This was followed by evaluation of cancellous bone in the distal femur metaphysis. For the femoral metaphysis, 42 consecutive slices (504 μm) of cancellous bone, 45 slices (540 μm) proximal to the growth plate/metaphysis boundary, were evaluated. We used irregular manual contouring a few pixels interior to the endocortical surface to delineate cancellous from cortical bone. Direct cancellous bone measurements included cancellous bone volume fraction (bone volume/tissue volume, %), connectivity density (mm^−3^), trabecular thickness (μm), trabecular number (mm^−1^), and trabecular separation (μm).

### Histomorphometry

Methods used for measuring bone histomorphometry have been described (28) with modifications for mice (29). Briefly, distal right femora were dehydrated in a graded series of ethanol and xylene, and embedded undecalcified in modified methyl methacrylate. A vertical bed microtome (Leica 2065) was used to cut coronal sections (4 μm thick), which were then affixed to slides precoated with 1% gelatin solution. One section/animal was stained for tartrate resistant acid phosphatase, counterstained with toluidine blue (Sigma, St. Louis), and used for cell-based measurements. All data were collected with a 20x objective using the OsteoMeasure System (OsteoMetrics, Inc., Atlanta, GA). The sampling site for the distal femoral metaphysis was located 0.25–1.25 mm proximal to the growth plate and 0.1 mm from cortical bone.

Cell-based measurements included osteoblast perimeter (osteoblast perimeter/bone perimeter, %), osteoclast perimeter (osteoclast perimeter/bone perimeter, %), marrow adipocyte area fraction (adipocyte area/tissue area, %), adipocyte density (number of adipocytes/tissue area, #/mm^2^) and adipocyte size (μm^2^). Osteoblasts were identified morphologically as plump cuboidal cells immediately adjacent to a thin layer of osteoid in direct contact with the bone perimeter. Osteoclasts were identified as multinucleated (two or more nuclei) cells with acid phosphatase positive (red-stained) cytoplasm in contact with the bone perimeter. Adipocytes were identified as large circular or oval-shaped cells bordered by a prominent cell membrane and lacking cytoplasmic staining due to alcohol extraction of intracellular lipids during processing. This method has been previously validated by fat extraction and analysis (30). All bone histomorphometric data are reported using standard 2-dimensional nomenclature (31).

### Gene Expression

Tibiae (*n* = 8/group) were pulverized with a mortar and pestle in liquid nitrogen and homogenized in Trizol (Life Technologies, Grand Island, NY). Total RNA was isolated according to the manufacturer's protocol, and mRNA was reverse transcribed into cDNA using SuperScript III First-Strand Synthesis SuperMix for qRT-PCR (Life Technologies). Gene expression for a panel of genes related to adipocyte differentiation and function (Mouse Adipogenesis RT2 Profiler PCR Array, PAMM-049ZE-4) was determined according to the manufacturer's protocol (Qiagen, Valencia, CA). Gene expression was normalized to GAPDH. Relative quantification was determined (ΔΔCt method) using RT2 Profiler PCR Array Data Analysis software version 3.5 (Qiagen). Fold-change was calculated relative to mice housed at 32°C as the reference control. Specifically, we compared changes in tibia gene expression in (1) mice housed at 22°C relative to mice housed at 32°C and (2) mice housed at 22°C and treated with propranolol relative to mice housed at 32°C to identify overlap between genes differentially expressed in response to sub-thermoneutral housing or propranolol. Furthermore, the expression of additional genes (*Adrb1, Adrb3, Alpl*, and *Bglap*) was determined using gene-specific primers (*Adrb1* - for: CTCATCGTGGTGGGTAACGTG, rev: ACACACAGCACATCTACCGAA; *Adrb3* – for: GGCCCTCTCTAGTTCCCAG, rev: TAGCCATCAAACCTGTTGAGC; *Alpl* – Qiagen RT^2^ qPCR Primer Assay PPM03155A; *Bglap*– Qiagen RT^2^ qPCR Primer Assay PPM04465F), and changes in gene expression between treatment groups were analyzed using the ΔΔCt method as described above.

### Statistical Analysis

A 2 × 2 factorial experimental design was used with mice randomized to a temperature group (22 or 32°C) and treatment group (vehicle control or propranolol). Mean comparisons were made using a two-factor linear model with an interaction between temperature and treatment. Residual analysis, Levene's test for homogeneity of variance, and Anderson-Darling tests of normality were used to assess the conditions for use of a linear model. A general linear model with unequal variances for the temperature groups, treatment groups, or both temperature and treatment groups (i.e., four distinct variance parameters) was used when the assumption of homogeneity of variance was violated. When interaction was present, inference focused on comparing temperature groups separately for vehicle and propranolol treated mice (i.e., focusing on how treatment modified the effect of temperature). The Benjamini and Hochberg (32) method for maintaining the false discovery rate at 5% was used to adjust for multiple comparisons. Differences were considered significant at *p* ≤ 0.05. All data are presented as mean ± SE. Data analysis was performed using R version 3.4.3.

## Results

**Figure 1:**
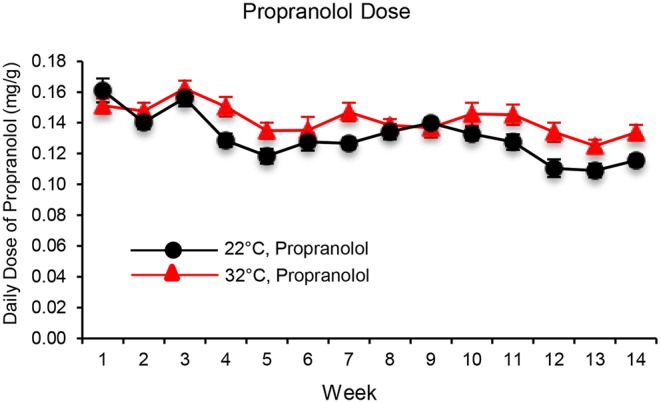
The daily dose of propranolol—administered in drinking water—in mice housed at room temperature (22°C) and thermoneutral temperature (32°C) over the 14-week duration of study. Data are mean ± SE. *N* = 10/group.

The daily dose of propranolol—administered in drinking water—in mice housed at room temperature (22°C) or thermoneutral temperature (32°C) is shown in Figure 1. When averaged over the 14 weeks of study, mice housed at 22 and 32°C consumed 0.13 ± 0.01 mg/g/d and 0.14 ± 0.01mg/g/d of propranolol, respectively.

**Figure 2:**
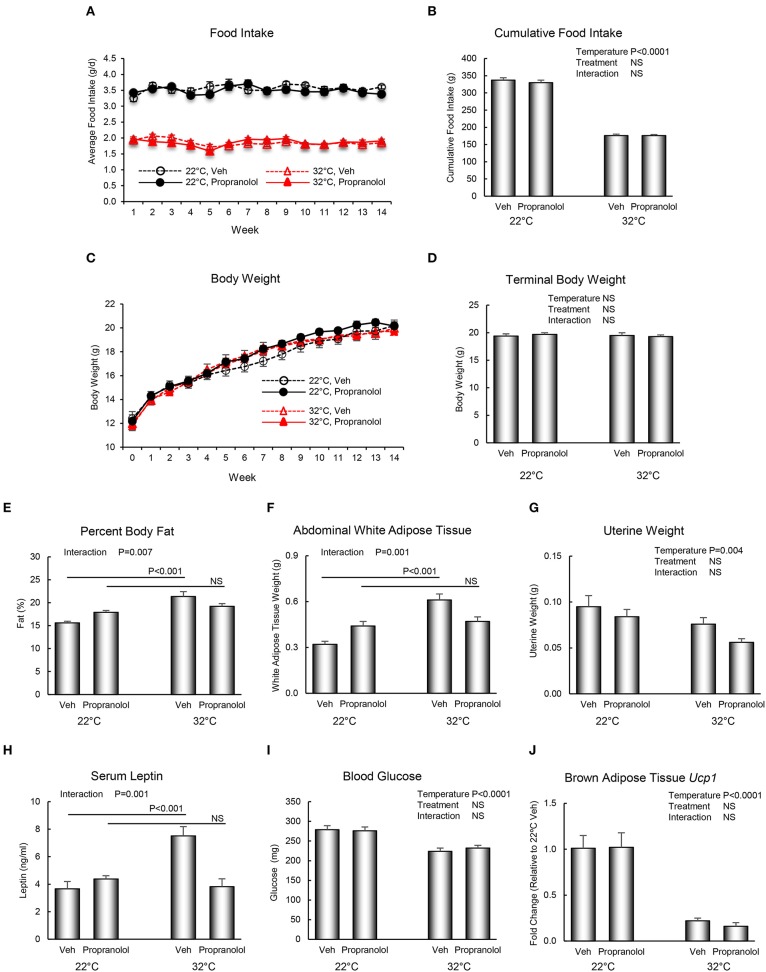
Effect of housing temperature (temperature) and treatment with propranolol (treatment) on **(A,B)** food intake, **(C,D)** body weight, **(E)** percent body fat, **(F)** abdominal white adipose tissue weight, **(G)** uterine weight, **(H)** serum leptin, **(I)** blood glucose, and **(J)** brown adipose tissue *Ucp1* gene expression. Data are mean ± SE. *N* = 10/group for **(A–I)** and *n* = 8/group for **(J)**. *Post–hoc* analysis was performed when there was a significant interaction term.

The respective effects of housing temperature and propranolol treatment on food and body weight, body composition, serum chemistry, and *Ucp1* gene expression in BAT are shown in Figure 2. Mice housed at room temperature consumed more food per day than mice housed at thermoneutrality (panel A), resulting in 91% increase in cumulative food intake (panel B). However, housing temperature had no effect on body weight (panels C and D). Treatment with propranolol had no effect on daily or cumulative food intake or on body weight and no housing temperature x treatment interactions were noted for the aforementioned endpoints. Interactions between housing temperature and treatment were detected for percent body fat (panel E), abdominal WAT weight (panel F), and serum leptin levels (panel H); treatment with propranolol prevented the differential effects of housing temperature on these endpoints. Room temperature-housed mice had higher uterine weight (panel G). Finally, room temperature-housed mice had higher blood glucose (panel I) and higher BAT *Ucp1* gene expression (panel J), endpoints not influenced by propranolol treatment.

**Figure 3:**
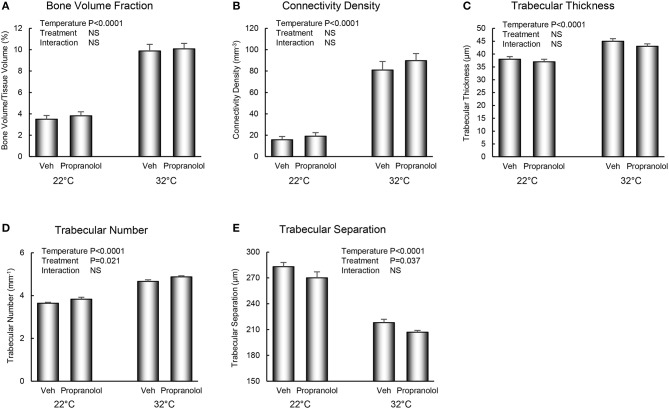
Effect of housing temperature (temperature) and treatment with propranolol (treatment) on cancellous bone architecture in distal femur metaphysic: **(A)** cancellous bone volume fraction, **(B)** connectivity density, **(C)** trabecular thickness, **(D)** trabecular number, and **(E)** trabecular separation. Data are mean ± SE. *N* = 10/group.

The respective effects of housing temperature and treatment with propranolol on bone microarchitecture in the distal femur metaphysis are shown in Figure 3. Mice housed at room temperature had lower bone volume fraction (panel A), connectivity density (panel B), trabecular thickness (panel C), and trabecular number (panel D), and higher trabecular separation (panel E). Treatment with propranolol resulted in higher trabecular number and lower trabecular separation. No housing temperature × treatment interactions were detected for any of the measured indices of bone microarchitecture.

**Figure 4:**
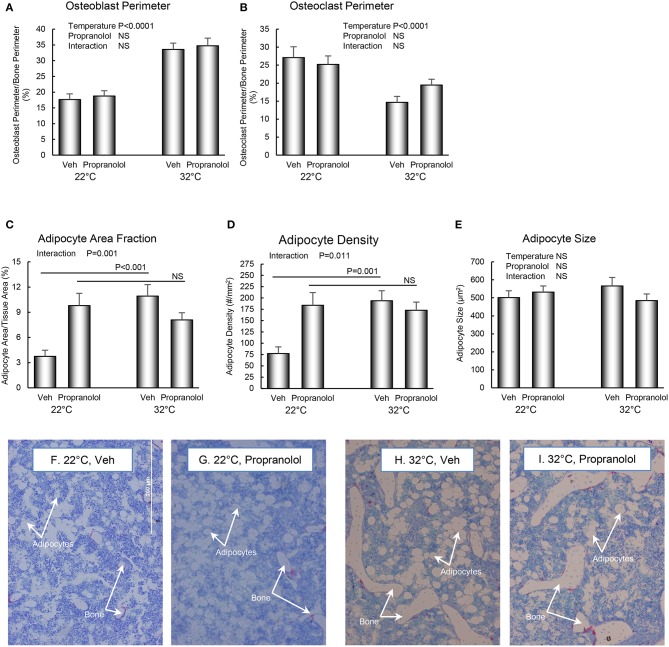
Effect of housing temperature (temperature) and treatment with propranolol (treatment) on indices of bone formation, bone resorption, and marrow adiposity in distal femur metaphysis: **(A)** osteoblast perimeter, **(B)** osteoclast perimeter, **(C)** adipocyte area fraction, **(D)** adipocyte density, and **(E)** adipocyte size. Representative images of the bone marrow compartment showing bMAT and cancellous bone in each treatment group in the distal femur metaphysis are shown in **(F–I)**. Data are mean ± SE. *N* = 10/group. *Post-hoc* analysis was performed when there was a significant interaction term.

The respective effects of housing temperature and treatment with propranolol on osteoblast perimeter, osteoclast perimeter and indices of bone marrow adiposity in distal femur metaphysis are shown in Figure 4. Mice housed at room temperature had lower osteoblast perimeter (panel A) and higher osteoclast perimeter (panel B). Interactions between housing temperature and treatment were detected for adipocyte area fraction (panel C) and adipocyte density (panel D); specifically, treatment with propranolol prevented the differential effects of housing temperature on both indices of marrow adiposity. However, neither housing temperature nor treatment altered adipocyte size. The differences in histomorphometry can be appreciated in the representative micrographs shown in panels F–I.

**Figure 5:**
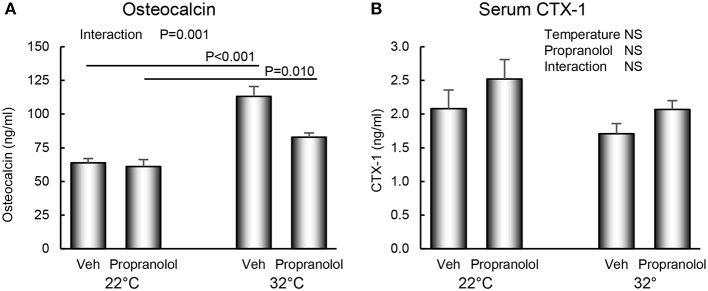
Effect of housing temperature (temperature) and treatment with propranolol (treatment) on **(A)** serum osteocalcin, a marker of global bone formation and **(B)** serum CTX-1, a marker of global bone resorption. Data are mean ± SE. *N* = 10/group. *Post-hoc* analysis was performed when there was a significant interaction term.

The respective effects of housing temperature and treatment with propranolol on serum osteocalcin and CTX-1 are shown in Figure 5. An interaction between housing temperature and treatment was noted for osteocalcin; the differential effects of housing temperature were attenuated by treatment with propranolol. Neither temperature nor treatment with propranolol had an effect on serum CTX-1.

**Figure 6:**
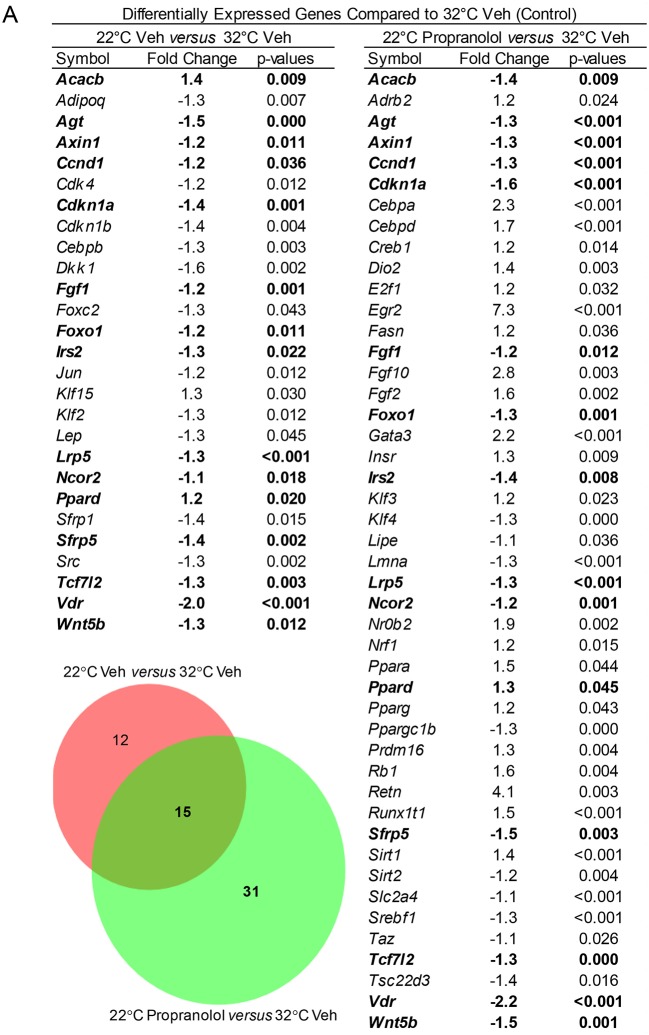
Effect of housing temperature and treatment with propranolol on expression of adipogenesis-associated genes (adipogenesis PCR array) in whole tibia. The Venn diagram shows the number of differentially expressed genes unique to temperature (22°C Veh vs. 32°C Veh, *n* = 12), number of differentially expressed genes unique to propranolol (22°C Propranolol vs. 32°C Veh, *n* = 31) and number shared by temperature and propranolol (*n* = 15). The gene list for differentially expressed genes is shown in the accompanying Table. Shared genes are bolded.

The respective effects of housing temperature and treatment on expression of a panel of 84 genes (adipogenesis PCR array), including *Adrb2* (β-adrenergic receptor 2), in tibia of mice housed at 22°C ± propranolol relative to mice housed at 32°C are shown in Figure 6. Housing temperature resulted in differential expression of 27 genes (22°C Veh vs. 32°C Veh); 12 of these genes were not differentially expressed in mice housed at 22°C and treated with propranolol and included genes for hormones (*Adipoq, Lep*), transcription factors and enhancer proteins (*Cebpb, Dkk1, Klf2, Klf15, Jun, Src*), regulators of cell cycle progression (*Cdk4, Cdkn1b, Foxc2*), and extracellular signaling factors (*Sfrp1*). Compared to mice housed at 32°C, *Acacb* was higher in mice housed at 22°C but lower in mice housed at 22°C and treated with propranolol. Finally, 31 differentially expressed genes were unique to propranolol treatment (22°C propranolol vs. 32°C vehicle). Expression levels for *Adrb2* did not differ between mice housed at 22 and 32°C. However, expression levels were higher in propranolol-treated mice housed at 22°C compared to vehicle-treated mice housed at 32°C.

In addition to the panel of 84 genes described above, we determined the effect of housing temperature and propranolol treatment on expression of two additional β-adrenergic receptor subtypes (*Adrb1* and *Adrb3*) and two genes coding for proteins related to bone formation, osteocalcin (*Bglap*) and alkaline phosphatase (*Alpl*). Mice housed at 22°C had lower levels of *Adrb3* (−1.8; *p* < 0.002), *Bglap* (−1.3; *p* < 0.03) and *Alpl* (−1.4; *p* < 0.003) compared to mice housed at 32°C but no difference in expression of *Adrb1*. Propranolol treatment of mice housed at 22°C did not influence expression of *Bglap, Alpl*, or *Adrb1*. Expression levels of *Adrb3* did not differ between propranolol-treated mice housed at 22°C and vehicle-treated mice housed at 32°C.

## Discussion

Compared to thermoneutral temperature (32°C), mice housed at room temperature (22°C) had lower percent body fat, lower abdominal WAT, lower cancellous bone volume fraction, connectivity density, trabecular thickness, trabecular number, osteoblast-lined bone perimeter, and bMAT (adipocyte area fraction and adipocyte density) in distal femur metaphysis, lower leptin and osteocalcin in serum, and lower mRNA levels for osteocalcin and alkaline phosphatase in tibia. In contrast, osteoclast-lined bone perimeter and trabecular separation were higher in room temperature-housed mice. Treatment with propranolol prevented housing temperature-associated differences in percent body fat, abdominal WAT, serum leptin, and distal femur metaphysis bMAT, but had no effect on bone response to housing temperature. Finally, there were shared as well as unique genes differentially regulated in response to room temperature housing and treatment with propranolol.

Housing of growing female and male mice at room temperature results in dramatic cancellous bone loss prior to skeletal maturity (5–7). The bone loss is not uniform, with more loss occurring in long bones than in lumbar vertebrae (7, 33) and in the distal femur, more loss occurring in metaphysis than in epiphysis (34). Importantly, housing mice at thermoneutrality prevents premature bone loss (6, 7). In female mice, changes in bMAT levels in response to differences in housing temperature follow a similar pattern in that the bMAT levels are lower in distal femur metaphysis of mice housed at room temperature (7). We did not measure bone marrow adiposity in lumbar vertebra or distal femur epiphysis because adipocytes are very uncommon at these sites in mice housed at either room or thermoneutral temperature. The lower levels of bMAT and lower levels of cancellous bone observed in female mice housed at room temperature appear consistent with a shared mechanism. However, a different response occurred in male mice; whereas premature cancellous bone loss was evident in males housed at room temperature, differences in bMAT were not observed (6). The divergent response of bMAT to room temperature-induced cold stress in female and male mice does not support a shared mechanism.

The quantity of bMAT in distal femur metaphysis is context-dependent and, as recently reviewed (35), influenced by numerous factors, including skeletal site, age, food availability, and housing temperature. Increased bMAT occurs with extremes in energy availability; obesity and rapid weight loss each result in increased bMAT. Treatment of rats with β-adrenergic agonists, such as isoproterenol, increased lipolysis in bMAT but to a lesser extent than inguinal WAT (36). Baek et al. (37) found that β-blockade using propranolol had no effect on bMAT in mice fed a normal diet but partially reduced increases in bMAT induced by both calorie restriction and calorie excess. Blocking β-adrenergic signaling in growing female rats fed a calorie-restricted diet attenuated reductions in circulating leptin, cancellous bone mass and increases in marrow adiposity. However, as was the case for mice fed a normal diet, propranolol treatment had no effect on bMAT in *ad libitum*-fed rats (19). Propranolol was shown to reduce dietary fat absorption and fat mass in mice fed high fat diet by suppressing expression of pancreatic lipase (26). In the present study, propranolol treatment prevented the differences in bMAT in distal femur metaphysis between female mice housed at room temperature and thermoneutral temperature.

We housed mice individually in the present study to facilitate measurement of food intake and to prevent huddling. Additionally, the mice were not offered material for nest building. These measures were important because mice employ huddling and nesting as strategies to decrease reliance on non-shivering thermogenesis when housed at sub-thermoneutral temperature (38). The 10°C higher housing temperature resulted in an impressive ~280% difference in cancellous bone volume fraction in distal femur metaphysis, a commonly evaluated skeletal site. While a detailed temperature response curve has yet to be established, the magnitude of observed change suggest even small differences in housing temperature, within or among vivariums, could influence results. Thus, factors with potential for altering thermoregulation (e.g., housing conditions or treatment) should be evaluated for their effect on bone mass, architecture, growth, and turnover.

The mechanisms mediating the impact of adaptive thermogenesis on bone metabolism during room temperature housing have received little attention. As anticipated, housing temperature strongly influenced gene expression levels for *Ucp1* in BAT but the adaptive response to changes in housing temperature was not altered by treatment with propranolol. This finding is consistent with evidence that activation of *Ucp1* gene expression by increased sympathetic outflow induced by cold stress is primarily mediated through β_3_ receptor signaling (39). Commonly referred to as a non-specific β-blocker, propranolol inhibits β_1_, β_2_ and β_3_ receptors. However, it is reported to be only a weak inhibitor of β_3_ at the dose used in the present study (40). This conclusion is supported by the lack of an effect of propranolol in the present study on *Ucp1* expression in BAT of mice housed at either room temperature or at thermoneutral. Notably, while propranolol treatment resulted in higher trabecular number in distal femur metaphysis, this effect was not influenced by temperature. Thus, it is unlikely that room temperature-induced bone loss requires β_1_ and β_2_ receptor signaling. It is interesting to note that room temperature housing resulted in lower mRNA levels for β_3_ receptor in tibia and that this response was blocked by treatment with propranolol. Further studies will be required to determine whether β_3_ receptor signaling contributes to bone loss in room temperature-housed mice.

Studies performed in *Ucp1* knockout mice suggest that *Ucp1* expression has a protective effect on bone during cold stress induced by sub-thermoneutral temperature housing (41). However, deletion of *Ucp1* increases activation of alternative forms of thermogenesis to maintain energy homeostasis, including increased shivering, in mice housed at room temperature. Because of the increased energy cost to activate alternative forms of adaptive thermogenesis, *Ucp1* knockout mice are resistant to diet-induced obesity when housed at room temperature. In contrast, when housed at thermoneutrality, *Ucp1* knockout mice have increased sensitivity to diet-induced obesity (42, 43). The fundamental difference in thermoregulation between mice and humans and the growing number of examples that housing temperature influences experimental outcomes (3, 44–59) argue strongly that thermoneutral housing of mice more accurately reflects the thermal environment in humans and preclinical studies performed in mice should be conducted at housing temperatures that minimize cold stress.

Propranolol treatment is associated with increased body weight in some individuals (22, 23, 60) and deletion of β_1_, β_2_, and β_3_ receptors in mice increases sensitivity to diet-induced weight gain (61). Adaptive thermogenesis in response to cold stress is energy demanding; in the present study, mice housed at room temperature required ~90% more energy to match the growth rate of mice housed at thermoneutrality. Although body weight did not differ, there were differences in body composition and serum leptin levels. Specifically, mice housed at room temperature had lower percent body mass as adipose tissue, lower abdominal WAT, and not surprisingly, lower serum leptin levels. Treatment with propranolol had no effect on weight but prevented the temperature-associated changes in body composition, potentially by inhibiting expression of pancreatic lipase as well as suppressing β-adrenergic signaling in bone.

Some, but not all, studies report that propranolol influences bone growth, mass and turnover in mice and rats. Factors such as gonadal status, mechanical loading and energy balance appear to influence the skeletal response to treatment with propranolol (18, 19, 21). In the present study, propranolol-treated mice exhibited small but significant changes in bone microarchitecture; trabecular number was increased and trabecular separation decreased. In contrast to adipose tissues, propranolol did not blunt the dramatic differences in bone mass, osteoblast-lined and osteoclast-lined bone perimeters, or expression levels for osteocalcin and alkaline phosphatase in long bones between mice housed at room temperature and thermoneutral temperature. These findings provide additional strong evidence that the mechanisms responsible for adipose and skeletal adaptations to cold stress differ. Elevated glucocorticoid and thyroid hormone levels, and decreased leptin levels may contribute to the rapid cancellous bone loss observed in male and female mice housed at room temperature. Glucocorticoids, thyroid hormone and leptin are important regulators of bone metabolism and their levels are altered by housing temperature (62–69). Further research is required to establish the role of changes in these hormones in premature cancellous bone loss in mice housed at sub-thermoneutral temperatures.

As previously indicated, sensory and sympathetic nerve fibers innervate bone (70, 71) and adrenoceptors, and receptors for vasointestinal peptide, substance P and calcitonin gene-related peptide are expressed on cells located within bone marrow, including osteoblasts, osteoclasts, chondrocytes, and their precursors (72–74). Cells within skeletal tissue also synthesize neuropeptides. Thus, cells in bone marrow are well positioned to respond to circulating as well as locally-produced neuropeptides whose levels are influenced by temperature. However, no change was observed in *Ucp1* gene expression in bone marrow (data not shown), a finding that contrasts with the increase in *Ucp1* gene expression in brown fat in room temperature-housed mice. Additionally, the decreases in cancellous bone mass and bone formation rate in room temperature-housed mice were associated with lower leptin, an adipokine known to increase sympathetic tone in mice (75–77). These findings do not preclude a role for sympathetic outflow in mediating the osteopenic effects of cold stress induced by room temperature housing but rather suggest they are unlikely to be mediated through β_1_ and β_2_ receptor signaling (78).

We evaluated expression levels for genes associated with adipogenesis and adipocyte function (using a PCR microarray), β-adrenergic receptors, and bone formation. These analyses identified genes in tibia of mice housed at room temperature in the presence or absence of propranolol that were differentially expressed compared to mice housed at thermoneutrality. These data reflect the product of number of cells within the tissue expressing a gene and expression level/cell and provide a broad-based index of the magnitude of change resulting from differences in housing temperature and treatment. In addition to genes exclusive to housing temperature (*n* = 12) and propranolol (*n* = 31), there were genes differentially expressed and changed in the same direction by both interventions (*n* = 14). One shared gene changed in opposite direction. Taken together, these findings further support the conclusion that β_1_ and/or β_2_ signaling contribute(s) to the changes in bMAT associated with adaptation to cold temperature stress. Based on our initial screen, it is notable that propranolol altered the effect of room temperature housing on expression levels of *Adipoq, Lep, Klf2*, and *Klf15*, genes whose protein products play important roles in adipogenesis (79–81). Specifically, these genes were no longer differentially expressed in response to room temperature housing. The expression level of the gene for acetyl-CoA carboxylase (*Acacb*), the rate-limiting step for fatty acid synthesis and key regulator of β-oxidation (82), was higher in mice housed at room temperature and lower in propranolol-treated mice, findings consistent with propranolol attenuating the lower bMAT in room temperature-housed mice. It is also of interest that propranolol treatment resulted in differential expression of a large number of genes not influenced by housing temperature. Bone marrow is a complex organ system and β-adrenergic receptors are not limited to mesenchymal cell lineages (83). Thus, the antagonistic effects of propranolol on the adaptive response of bMAT to changes in housing temperature could be due a combination of direct effects on β-adrenergic signaling by adipocyte progenitors and/or adipocytes and indirect effects mediated through β-adrenergic signaling by cells not related to adipocytes (e.g., hematopoietic lineage cells). Additional research is required to decipher the precise contribution of target cells and individual signaling pathways.

In summary, the lower percent body fat and abdominal WAT weight and higher *Ucp1* gene expression in BAT in room temperature-housed mice, compared to mice housed at thermoneutrality, is consistent with increased β-oxidation and increased non-shivering thermogenesis, respectively. Both mechanisms likely contribute to the temperature-associated differences in body composition. Our findings evaluating the effects of propranolol on mice housed at room temperature or thermoneutral suggest that β_1_/β_2_ receptor signaling contributes to lower WAT and bMAT levels in room temperature-housed mice. Osteoblasts and adipocytes share a common mesenchymal stem cell progenitor (84). However, failure of propranolol to attenuate the reduction in osteoblast-lined bone perimeter associated with room temperature cold stress does not support β_1_/β_2_ receptor signaling as a mechanism for premature bone loss associated with standard room temperature housing.

## Data Availability Statement

All data for this study are included in the article. The datasets will also be deposited with NASA and made available to anyone upon request.

## Author Contributions

RT, KP, AB, and UI: Conceptualization. KP, CW, AG, and RT: Data collection. AB: Data analysis. RT: Drafting manuscript. RT, KP, CW, AB, AG, and UI: Revising manuscript content. RT, KP, CW, AB, AG, and UI: Approving final version. UI: Takes responsibility for the integrity of the data.

### Conflict of Interest

The authors declare that the research was conducted in the absence of any commercial or financial relationships that could be construed as a potential conflict of interest.
